# Moderate alcohol consumption and clinical outcomes in MASLD: a systematic review and meta-analysis of longitudinal cohorts

**DOI:** 10.1186/s12876-026-04814-5

**Published:** 2026-04-30

**Authors:** Neilson Silveira de Souza, Helma Pinchemel Cotrim, Antônio Ricardo Cardia Ferraz de Andrade

**Affiliations:** Faculty of Medicine of Bahia, Federal University of Bahia, Salvador, Bahia, Brazil

**Keywords:** Metabolic Dysfunction-Associated Steatotic Liver Disease, Alcohol, Hepatocellular Carcinoma, Mortality, Fibrosis

## Abstract

**Background and aim:**

Metabolic Dysfunction-Associated Steatotic Liver Disease (MASLD) remains controversial regarding the impact of moderate alcohol consumption on disease progression and clinical outcomes. This systematic review aimed to evaluate the effects of moderate alcohol intake (up to 30 g/day for men and 20 g/day for women) on the development and progression of Steatotic Liver Disease (SLD), including outcomes such as histopathological progression, hepatocellular carcinoma (HCC), cardiovascular disease, and mortality.

**Methods:**

The review followed PRISMA guidelines and included longitudinal cohort studies identified through PubMed, Scopus, Web of Science, Embase, and LILACS. Nine studies were selected for qualitative analysis, and seven were included in the meta-analysis. Effect measures, Hazard Ratios (HR) and Odds Ratios (OR), were pooled using Cochrane RevMan, and study quality was assessed with the Newcastle–Ottawa Scale. PROSPERO: CRD420261277466.

**Results:**

The meta-analysis showed no statistically significant association between moderate alcohol consumption and fibrosis progression (HR: 1.63 [95% CI: 0.96–2.77]; OR: 1.44 [95% CI: 0.56–3.72]). However, a statistically significant protective association was observed for all-cause mortality (HR: 0.73 [95% CI: 0.62–0.86]). High heterogeneity was noted across studies (I^2^ = 56.9–86.2%). Excessive alcohol consumption remained a strong risk factor for adverse outcomes, and the incidence of HCC significantly increased among patients with advanced fibrosis, even with light alcohol intake.

**Conclusions:**

Even moderate alcohol consumption may contribute to the progression of severe hepatic outcomes in SLD. These findings support clinical recommendations for total abstinence, particularly in patients with established fibrosis, to prevent progression to cirrhosis and hepatocellular carcinoma.

**Supplementary Information:**

The online version contains supplementary material available at 10.1186/s12876-026-04814-5.

## Introduction

Metabolic Dysfunction-Associated Steatotic Liver Disease (MASLD) is one of the most prevalent causes of chronic liver disease, affecting approximately 38% of the global adult population. This condition is intrinsically linked to the global epidemics of obesity, type 2 diabetes, and metabolic syndrome, with projections indicating a continuous rise in its incidence over the coming decades [1].

Evidence demonstrates a synergistic effect between alcohol consumption and metabolic syndrome, where high alcohol intake accelerates the progression of hepatic fibrosis in individuals with obesity or diabetes [2]. This scenario led to the creation of a new diagnostic category, MetALD, which identifies patients with MASLD who consume higher levels of alcohol, acknowledging their increased risk of severe liver disease [3].

However, this interaction between alcohol and metabolic syndrome creates a significant dilemma in clinical guidance: should total alcohol abstinence be recommended, or can the patient consume alcohol moderately? Major international guidelines, such as those from the American Association for the Study of Liver Diseases (AASLD) and the European Association for the Study of the Liver (EASL), strongly recommend alcohol abstinence for patients with MASLD, especially in the presence of fibrosis, as there is no proven safe threshold for consumption [3, 4]. Yet, this recommendation contrasts with the public and previously medical perception that light to moderate alcohol consumption could confer a protective effect [5]. The lack of a clear consensus on safe limits and conflicting health messages hinders effective clinical counseling, leaving both patients and physicians without definitive guidance.

The rationale for this study is based on the need for a deeper understanding of alcohol's influence on liver health. Such knowledge is crucial for guiding prevention and treatment strategies, as well as providing the foundation for more precise and personalized clinical recommendations for the general population.

The objective of this work is to conduct a systematic literature review to synthesize evidence on how moderate alcohol consumption influences the risk of developing advanced liver disease and complications in patients diagnosed with steatotic liver disease. By doing so, we expect to provide a solid evidence base to guide clinical counseling in primary and specialized care, aiming to prevent severe hepatic outcomes in this vulnerable and growing population.

## Methods

### Overview

This systematic review was conducted in accordance with the recommendations of the Preferred Reporting Items for Systematic Reviews and Meta-Analyses (PRISMA). The search was performed on June 27, 2024. The systematic review protocol was registered with PROSPERO (CRD420261277466) and was developed, reviewed and approved by all authors. No amendments were made to the information provided at registration.

### Databases and search strategy

A comprehensive literature search was conducted across the PubMed, Scopus, Web of Science, Embase, and LILACS databases for studies published between January 1, 1980, and June 27, 2024. Language was restricted to English. In addition to database searching, we performed a manual search of the reference lists of all included studies and relevant prior reviews to identify potentially eligible records not captured by the electronic search.

The search strategy combined descriptors related to steatotic liver disease and alcohol consumption, utilizing the following terms: ("fatty liver" OR "steatotic liver" OR "hepatic steatosis" OR "nonalcoholic fatty liver disease" OR MASLD OR NAFLD OR steatohepatitis) AND ("alcohol consumption" OR "alcohol intake" OR "mild drinking" OR "light drinking" OR "low alcohol consumption" OR "light alcohol consumption" OR "moderate alcohol consumption" OR "moderate drinking"). Gray literature sources were not systematically searched.

### Eligibility criteria

This systematic review included prospective or longitudinal cohort studies involving adult individuals (age ≥ 18 years) with a diagnosis of MASLD. The central exposure criterion was the assessment of alcohol consumption as a variable of interest, specifically focusing on levels considered moderate compared to abstinent patients. Moderate alcohol consumption was defined as up to 30 g/day or 210 g/week for men, and up to 20 g/day or 140 g/week for women, consistent with AASLD guidelines [4]. Further details regarding the inclusion criteria are provided in Table 1. Articles that did not qualify as original research were excluded. Studies focusing exclusively on pediatric populations or patients with other primary liver diseases, where MASLD was not the primary focus, were also discarded. Detailed exclusion criteria are presented in Table 1.

**Table 1:** PICO-based eligibility criteria for studies on moderate alcohol use and MASLD outcomes

Component	Inclusion Criteria	Exclusion Criteria
Population	Adult individuals (age ≥ 18 years) diagnosed with MASLD. Population-based, community-based, or clinical cohort studies	Pediatric populations. Patients with other primary liver diseases (e.g., viral hepatitis, autoimmune liver disease, cirrhosis from causes other than MASLD)
Intervention	Moderate alcohol consumption (up to 30 g/day or 210 g/week for men; up to 20 g/day or 140 g/week for women) per AASLD guidelines [4]	Studies assessing only heavy alcohol consumption. Studies that did not quantify or categorize alcohol intake
Comparison	Comparison between different levels of alcohol consumption (e.g., abstainers vs. moderate consumers)	Studies without a clear comparison group or risk analysis related to consumption quantity/pattern
Outcome	Measurement of hepatic outcomes, such as fibrosis progression. Incidence of clinical outcomes including hepatocellular carcinoma, cardiovascular events, and mortality	Studies that did not report specific hepatic, cardiovascular, or all-cause mortality outcomes
Study Design	Cohort studies (prospective or retrospective)	Cross-sectional studies, randomized clinical trials, reviews (narrative, systematic, or meta-analyses), case reports, case series, and editorials

### Study selection

Based on PRISMA guidelines, study selection was performed in three stages: (1) removal of duplicates, (2) screening of titles and abstracts, and (3) full-text review of studies that met the previous criteria. Screening was conducted online using the Catchii platform [6].

All retrieved records were imported into the Catchii systematic review platform for deduplication and screening. Study selection was conducted using a dual-independent reviewer approach. Two authors independently screened titles and abstracts to identify potentially eligible studies, followed by independent full-text assessment based on predefined inclusion and exclusion criteria. Discrepancies between authors were resolved by consensus together with a third author. This process was implemented to minimize selection bias and enhance the methodological rigor and standardization of the study selection procedure, in accordance with PRISMA guidelines.

### Risk of bias and study quality

The Newcastle–Ottawa Scale (NOS) was used to evaluate the quality of the included studies. The NOS is a validated tool recommended for assessing non-randomized observational studies, such as the cohort studies selected for this review. The scoring system assigns a "star" for each quality criterion met, with a maximum of nine stars. Studies were categorized based on their final score: 7 to 9 stars (high quality/low risk of bias), 4 to 6 stars (moderate quality), and 0 to 3 stars (low quality/high risk of bias).

### Data extraction

A tailored data extraction form was developed for this study, incorporating the variables of interest required to address the research question. For each selected article, the following information was extracted: study identification (author, year), country of origin, study design (prospective or retrospective), sample size, demographic characteristics (age and sex distribution), the definition of moderate alcohol consumption used by the authors, the method of alcohol intake assessment (e.g., questionnaire, biomarker), follow-up duration (years), primary statistical results reported (e.g., Hazard Ratio, Odds Ratio), and the specific outcomes evaluated. The extracted data were compiled into a summary table to facilitate comparative analysis and qualitative synthesis of the evidence.

In addition, sensitivity analyses were conducted for outcomes with enough studies to evaluate the robustness of the pooled estimates. A leave-one-out approach was used, whereby each study was sequentially removed to assess its impact on the overall results and to identify potential sources of heterogeneity.

### Data synthesis and analysis

To quantify the effect of moderate alcohol consumption on the risk of liver disease progression, a meta-analysis was performed using Cochrane’s RevMan statistical software. The selected effect measures were Hazard Ratios (HR) and Odds Ratios (OR) with their respective 95% confidence intervals (95% CI), extracted from cohort studies comparing a "moderate consumption" group to an "abstinence" reference group. For data pooling, a random-effects model was employed, assuming that studies from diverse populations and contexts estimate distinct true effects.

Heterogeneity between studies was assessed using the I^2^ statistic, and results were presented graphically through forest plots and funnel plots, with publication bias assessed through visual inspection of funnel plots and quantitatively evaluated using Egger’s linear regression test for meta-analyses including at least three studies. For analyses including fewer than three studies, publication bias assessment was not performed due to insufficient statistical power.

## Results

### Study selection

**Fig. 1:**
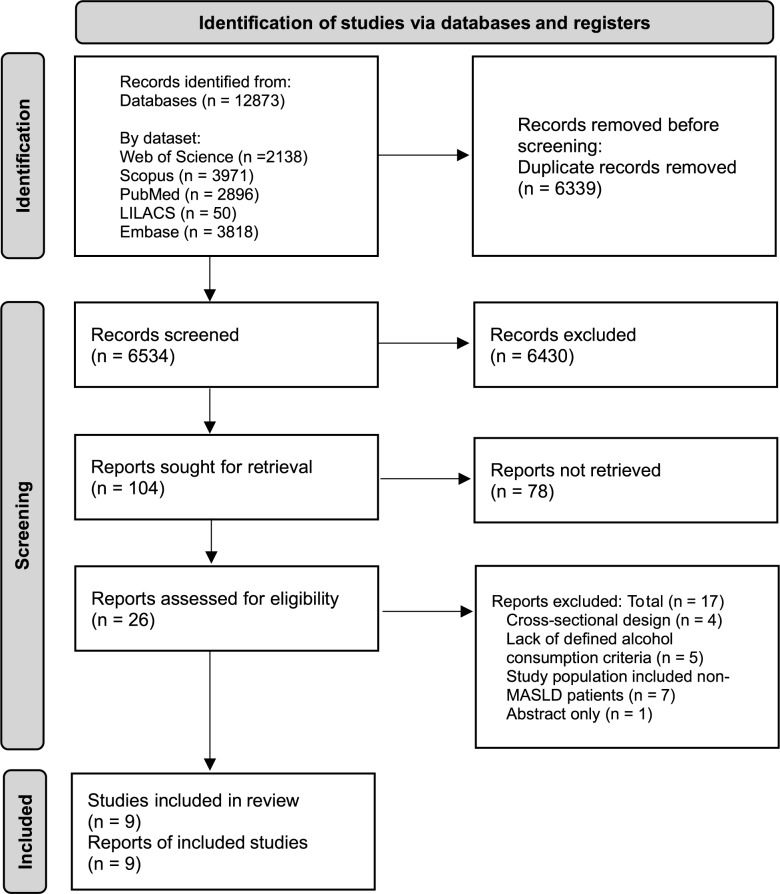
PRISMA flow diagram of the study selection process

The study search and selection process is detailed in the PRISMA flowchart (Fig. 1). The initial search in the Web of Science, Scopus, PubMed, LILACS, and Embase databases identified 12,873 records. After removing 6,339 duplicate articles, 6,534 studies underwent title and abstract screening. At this stage, 6,430 articles were excluded for not meeting the eligibility criteria.

Of the remaining 104 articles selected for full-text review, 78 could not be retrieved. The 26 articles assessed in full were evaluated for eligibility, and 17 were excluded for the following reasons: cross-sectional study design (*n* = 4), lack of clear criteria for alcohol consumption (*n* = 5), study population including patients without MASLD (*n* = 7), and publication available only as an abstract (*n* = 1). At the end of the process, 9 studies met all inclusion criteria and were included in the qualitative analysis of this systematic review.

### General study characteristics

A total of nine studies were selected; five addressed the impact of alcohol consumption on hepatic histopathology [7–11], while four focused on clinical impacts, including cardiovascular disease, hepatocellular carcinoma (HCC), and mortality [12–15]. The publication period for these articles ranged from 2009 to 2023. In total, 77,502 patients were included, with a mean cohort follow-up duration of 11.79 years. Regarding study design, eight were prospective [7–13, 15] and one was retrospective [14]. Further details regarding the general characteristics of the studies are presented in Table 2 and Table 3.

**Table 2:** General characteristics of the studies evaluating histopathological progression

Reference	Country	Follow-up (Years)	Participants (*n*)	Mean age (Years)	Sex (% Male)	Definition of Moderate Alcohol Consumption	Alcohol Assessment Method	Evaluated Outcome
Åberg et al., 2019 [7]	Finland	11.1	8,345	53.7	60.5	Up to 20 g/day (M and F)	Questionnaire	Fibrosis progression and Mortality
Ajmera et al., 2018 [8]	USA	3.9	285	47	30.2	Up to 20 g/day (M and F)	AUDIT and Skinner questionnaires	Fibrosis progression
Blomdahl et al., 2023 [9]	Sweden	17.2	82	47.3	72	< 140 g/week (M and F)	Interview, AUDIT, and PEth biomarker	Fibrosis progression
Chang et al., 2019 [10]	South Korea	8.3	58,927	37.7	82	1–30 g/day (M); 1–20 g/day (F)	Questionnaire	Fibrosis progression
Ekstedt et al., 2009 [11]	Sweden	13.8	71	47.3	72	≤ 140 g/week (M and F)	Questionnaire (AUDIT) and interview	Fibrosis progression

*Abbreviations*: *AUDIT* Alcohol Use Disorders Identification Test, *CI* confidence interval, *F* female, *HR* hazard ratio, *M* male, *OR* odds ratio, *PEth* phosphatidylethanol

**Table 3:** General characteristics of the studies evaluating clinical outcomes

Reference	Country	Follow-up (Years)	Participants (*n*)	Mean age (Years)	Sex (% Male)	Definition of Moderate Alcohol Consumption	Alcohol Assessment Method	Evaluated Outcome
Hajifathalian et al., 2019 [12]	USA	5.8	4,568	48	44.1	7–20 g/day (F); 7–30 g/day (M)	Questionnaire	Mortality
Janjua et al., 2022 [13]	Australia	20	659	56.9	58.3	14–98 g/week (M and F)	Questionnaire	Cardiovascular complication
Kimura et al., 2018 [14]	Japan	6	301	56	45	Up to 20 g/day (M and F)	Questionnaires/interviews	Hepatocellular Carcinoma (HCC)
Younossi et al., 2019 [15]	USA	20	4,264	45.9	51	Up to 30 g/day (M); Up to 15 g/day (F)	Questionnaire	Mortality

*Abbreviations*: *AUDIT* Alcohol Use Disorders Identification Test, *CI* confidence interval, *F* female, *HR* hazard ratio, *M* male, *OR* odds ratio

The methodology for assessing alcohol consumption and the definitions of "moderate consumption" varied substantially across the studies. Most utilized self-administered questionnaires to quantify intake. Several studies specified the use of validated tools, such as the Alcohol Use Disorders Identification Test (AUDIT), Skinner questionnaires, or the AUDIT-C [8, 9, 11], sometimes combined with interviews. Notably, one study complemented subjective methods with the blood biomarker Phosphatidylethanol (PEth) for a more objective assessment of alcohol intake [9].

The definition of "moderate consumption" was particularly heterogeneous. Some studies defined a threshold of up to 20 g/day for both men and women [7, 8, 14]. Others established different limits for men (1–30 g/day or < 30 g/day) and women (1–20 g/day or < 20 g/day) [10, 12]. Definitions based on weekly consumption were also used, such as < 140 g/week for both sexes [9, 11].

### Quality and risk of bias assessment

The methodological quality of the nine studies was rated as "high" according to the Newcastle–Ottawa Scale (NOS) (Table 4), with scores ranging between 8 and 9 out of a maximum of 9 stars, indicating a low overall risk of bias. The primary strengths of these studies include robust control for confounding factors (such as age, BMI, and diabetes) and the use of hard outcomes, such as mortality confirmed by national registries or fibrosis progression assessed by liver biopsy.

**Table 4:** Risk of bias assessment of cohort studies using the Newcastle–Ottawa Scale (NOS)

Study	Selection				Comparability		Outcome			Total
	S1	S2	S3	S4	C1	C2	O1	O2	O3	
Åberg et al., 2019 [7]	*	*	*	*	*	*	*	*	*	9
Ajmera et al., 2018 [8]	*	*	*	*	*	*	*	*	*	9
Blomdahl et al., 2023 [9]	*	*	*	*	*	*	*	*	*	9
Chang et al., 2019 [10]	*	*	*	*	*	*	*	*	0	8
Ekstedt et al., 2009 [11]	*	*	*	*	*	*	*	*	*	9
Hajifathalian et al., 2019 [12]	*	*	*	*	*	*	*	*	*	9
Janjua et al., 2022 [13]	*	*	*	*	*	*	*	*	*	9
Kimura et al., 2018 [14]	*	*	*	*	*	*	*	*	*	9
Younossi et al., 2019 [15]	*	*	*	*	*	*	*	*	*	9

*Abbreviations*: (*S*) S1: Representativeness, S2: Non-exposed cohort selection, S3: Exposure ascertainment, S4: Absence of outcome at baseline. Comparability (C) C1: Control for primary factor; C2: Control for additional factors. Outcome (O): O1: Assessment; O2: Adequate follow-up duration; O3: Adequate follow-up rate

The main limitation identified was the reliance on self-reported questionnaires to measure alcohol consumption, a method prone to underreporting. However, the study by Blomdahl et al. (2023) [9] minimized this risk by incorporating the objective biomarker PEth. Despite this limitation, the body of evidence in this review is considered methodologically strong.

Visual inspection of funnel plots for all-cause mortality and fibrosis progression (OR) did not suggest evident asymmetry. This was supported by Egger’s regression test, which did not indicate significant publication bias for all-cause mortality (HR) (intercept = 0.02, 95% CI: − 3.82 to 3.85; *p* = 0.995) or fibrosis progression (OR) (intercept = 1.74, 95% CI: − 1.87 to 5.36; *p* = 0.518). Publication bias was not assessed for fibrosis progression (HR) due to the limited number of studies (*n* = 2). Funnel plots are presented in the Supplementary Material (Figure S1).

### Risk of liver fibrosis progression

**Fig. 2:**
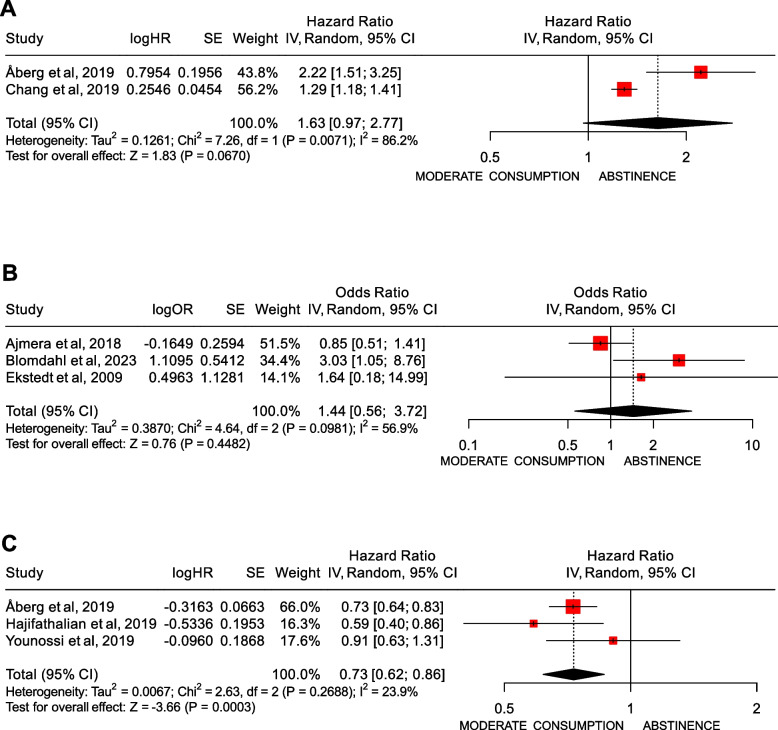
Hazard Ratio (**A**) and Odds Ratio (**B**) for the risk of liver fibrosis progression, and Hazard Ratio (**C**) for the risk of mortality in patients with steatotic liver disease with moderate alcohol consumption compared to abstinent patients

The meta-analysis was conducted to evaluate the risk of liver fibrosis progression in moderate alcohol consumers compared with abstainers [7–11]. Figure 2A presents the pooled results of studies that used Hazard Ratios [7, 10]. The combined analysis showed a Hazard Ratio (HR) of 1.63, suggesting a 63% increased risk of fibrosis progression in the moderate consumption group. However, this result did not reach statistical significance, as the 95% confidence interval [0.97; 2.77] included the null value (1.0). The lack of statistical significance is explained by the high heterogeneity observed between the studies (I^2^ = 86.2%), indicating substantial differences in their findings.

Sources of heterogeneity include differences in alcohol assessment methods (ranging from self-reported questionnaires to objective biomarkers such as phosphatidylethanol [PEth]), variability in study populations, and inconsistent definitions of moderate alcohol consumption (20–30 g/day). These factors contributed to wide confidence intervals and variability in individual study estimates.

The meta-analysis shown in Fig. 2B evaluated the Odds Ratio (OR) for liver fibrosis progression, comparing moderate alcohol consumers with abstinent patients [8, 9, 11]. The pooled result yielded an Odds Ratio of 1.44, indicating a 44% increased likelihood of fibrosis progression in the moderate consumption group. Nevertheless, the overall result was not statistically significant, as the 95% confidence interval [0.56; 3.72] was wide and crossed the null value (1.0). The heterogeneity analysis (I^2^ = 56.9%) demonstrated moderate to substantial variability among studies, which explains the imprecision of the pooled estimate. This variability is reflected in the individual study results, where only Blomdahl et al. (2023) found a statistically significant association, while the other two studies did not demonstrate a clear effect.

### Risk of hepatocellular carcinoma (HCC)

In the study by Kimura et al. (2018) [14], the impact of light alcohol consumption (defined as less than 20 g/day) on the risk of developing Hepatocellular Carcinoma (HCC) was investigated in patients with Nonalcoholic Fatty Liver Disease (NAFLD). The analysis showed that the incidence of HCC was significantly higher in the light alcohol consumption group compared with abstainers (6.5% vs. 1.4%).

Although the analysis of all patients with MASLD demonstrated that advanced fibrosis and diabetes mellitus were the main predictors of HCC, alcohol consumption had only a marginal overall effect. However, when specifically analyzing the subgroup of patients who already had advanced fibrosis (stages F3–F4), light alcohol intake emerged as a significant and independent risk factor for HCC development, increasing the risk nearly fivefold (Hazard Ratio: 4.83).

The study concluded that alcohol consumption, even at light levels, represents a risk factor for hepatic carcinogenesis in patients with NAFLD, particularly among those with pre-existing advanced fibrosis.

### Risk of cardiovascular complications

The study by Janjua et al. (2022) [13] analyzed the association between alcohol consumption and cardiovascular outcomes in a population-based cohort of 659 patients with MASLD followed for 20 years. The main finding was that low to moderate alcohol consumption was associated with a lower risk of hospitalization for cardiovascular disease (CVD), but not with reduced cardiovascular mortality.

Specifically, individuals who consumed 8 to 21 drinks per week had a 29% lower risk of CVD-related hospitalization compared with those who consumed 1 to 7 drinks per week (Hazard Ratio [HR] 0.71). Sex-stratified analysis showed that men who consumed between 8 and 21 drinks per week had a 38% lower risk of CVD hospitalization [13].

However, the study emphasized that this protective association was not observed in individuals with a binge drinking pattern (heavy episodic drinking). Furthermore, no significant association was found between different levels of alcohol consumption and cardiovascular mortality [13].

### All-cause mortality

In Fig. 2C, the meta-analysis pooled results from three cohort studies [7, 12, 15] to evaluate the association between moderate alcohol consumption and all-cause mortality in patients with steatotic liver disease. Individually, the studies by Åberg et al. [7] (HR 0.73; 95% CI: 0.64–0.83) and Hajifathalian et al. [12] (HR 0.59; 95% CI: 0.40–0.86) demonstrated a statistically significant reduction in mortality risk. The study by Younossi et al. showed a similar trend but did not reach statistical significance (HR 0.91; 95% CI: 0.63–1.31).

The pooled meta-analysis result demonstrated a statistically significant protective effect. Overall, moderate alcohol consumption was associated with a 27% reduction in all-cause mortality risk compared with abstinence, with a combined Hazard Ratio (HR) of 0.73 (95% CI: 0.62–0.86). The high statistical significance of this finding was confirmed by a p-value of 0.0003.

Additionally, the heterogeneity analysis showed low inconsistency among the studies (I^2^ = 23.9%; *p* = 0.2688), supporting the validity of combining their results and strengthening the overall conclusion of the analysis.

### Sensitivity analysis of pooled estimates

Sensitivity analyses using a leave-one-out approach were conducted to assess the robustness of the pooled estimates and to identify potential sources of heterogeneity. For liver fibrosis progression using Hazard Ratios (HR), exclusion of Åberg et al. resulted in a statistically significant association (HR: 1.29; 95% CI: 1.18–1.41), whereas exclusion of Chang et al. increased the pooled estimate (HR: 2.22; 95% CI: 1.51–3.25), indicating that individual studies had a notable influence on the magnitude of effect [7].

In the leave-one-out sensitivity analysis using Odds Ratios (OR), the exclusion of Ajmera et al. resulted in a statistically significant association between moderate alcohol consumption and fibrosis progression (OR: 2.70; 95% CI: 1.04–7.04; *p* = 0.0415), accompanied by complete elimination of heterogeneity (I^2^ = 0%). This finding suggests that Ajmera et al. was a major contributor to between-study variability and that, in its absence, the remaining studies demonstrated a more consistent and homogeneous harmful effect of moderate alcohol consumption on fibrosis progression [8].

Overall, these findings indicate that while the magnitude and statistical significance of fibrosis progression estimates are sensitive to individual studies, the direction of effect remains consistently toward increased risk.

## Discussion

### General interpretation of findings

The findings of this systematic review suggest a potential association between moderate alcohol consumption and liver fibrosis progression in patients with MASLD. However, this association did not reach statistical significance in pooled meta-analyses, as reflected by confidence intervals crossing the null value.

Despite this, the direction of effect across several longitudinal studies was generally consistent toward an increased risk of fibrosis progression. Individual studies reported risk estimates ranging from a 29% increase (HR 1.29) [10] to more than a twofold increase (HR 2.22; 95% CI: 1.51–3.25; p < 0.001) [7] among moderate drinkers compared with abstainers. Moreover, studies incorporating objective measures of alcohol exposure, such as phosphatidyl ethanol (PEth), demonstrated even stronger associations, with over a threefold increase in the risk of histopathological progression (OR 3.03; 95% CI: 1.05–8.76; *p* = 0.041) [8].

Taken together, these findings indicate that although low-to-moderate alcohol consumption has been associated with reduced cardiovascular risk and lower all-cause mortality in certain populations, it remains a clinically relevant and potentially modifiable driver of liver disease progression [16]. This effect appears particularly pronounced in individuals with underlying metabolic dysfunction or pre-existing fibrosis, in whom even modest alcohol intake may exert a synergistic deleterious effect, accelerating progression to cirrhosis and hepatocellular carcinoma [17]. In line with emerging evidence highlighting prognostic factors of tumor aggressiveness, such as lymphatic vessel density and immune-related pathways, and the evolving complexity of therapeutic strategies in hepatocellular carcinoma, the cumulative burden of alcohol-related liver injury further reinforces an unfavorable hepatic milieu [18–20]. Therefore, within the context of MASLD management, any potential systemic benefits of moderate alcohol consumption are outweighed by its liver-specific risks.

### The absence of a safe threshold

From a pathophysiological standpoint, the lack of a safe threshold for alcohol consumption can be explained by the activation of oxidative pathways, particularly the induction of cytochrome P450 2E1 (CYP2E1) [21]. Ethanol-mediated upregulation of CYP2E1 promotes the generation of reactive oxygen species (ROS) and the accumulation of acetaldehyde, a highly toxic metabolite that induces mitochondrial dysfunction and hepatocyte apoptosis. In addition, alcohol enhances hepatic de novo lipogenesis and stimulates the release of pro-inflammatory cytokines, including tumor necrosis factor-α (TNF-α) and interleukin-6 (IL-6), thereby exacerbating the chronic inflammatory milieu characteristic of MASLD [22]. In individuals with metabolic syndrome, this interaction is particularly deleterious, as alcohol amplifies lipotoxicity and inflammatory signaling driven by insulin resistance [23].

Accordingly, the findings of this review are consistent with recent clinical practice guidelines from the American Association for the Study of Liver Diseases (AASLD, 2023) and the European Association for the Study of the Liver (EASL, 2024), which underscore that no level of alcohol consumption can be considered safe for patients with steatotic liver disease. Even alcohol intake traditionally classified as “light” may contribute to fibrosis progression and increase the risk of long-term hepatic complications. These data support a conservative clinical approach, favoring the recommendation of complete alcohol abstinence in patients with MASLD, particularly in those with metabolic risk factors or established advanced fibrosis [3, 4].

### Apparent protective effect on mortality

**Fig. 3:**
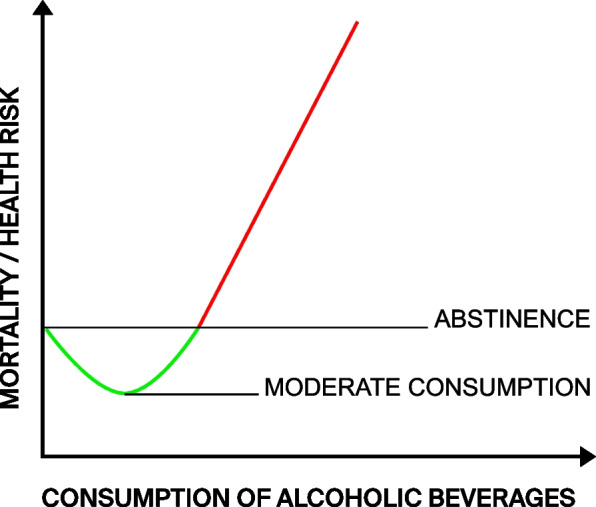
J-shaped curve illustrating the relationship between alcohol consumption and health risk. The graph depicts the apparent protective effect of moderate intake on all-cause mortality compared to abstinence, followed by a sharp escalation in health risks, such as liver fibrosis and malignancy, as alcohol consumption increases

One of the most notable findings of this review was the observed association between light-to-moderate alcohol consumption and lower all-cause mortality among patients with MASLD [7, 12, 15]. Several mechanisms have been proposed to explain this association. The most widely cited hypothesis relates to a potential cardioprotective effect of low alcohol intake, including increases in high-density lipoprotein (HDL) cholesterol, improvements in insulin sensitivity, and attenuation of systemic inflammation, which together may reduce cardiovascular risk [15]. However, these effects appear to be confined to a narrow range of consumption and are lost with higher intake levels, in line with the “J-shaped” relationship between alcohol consumption and mortality described in epidemiological studies (Fig. 3) [24].

Importantly, the apparent reduction in all-cause mortality may partly reflect methodological limitations rather than a true protective effect. A key concern is misclassification bias within the reference group, as many studies compare moderate drinkers with a heterogeneous group of “abstainers” [25]. This category often includes former drinkers who stopped consuming alcohol because of underlying illness or declining health, a phenomenon commonly referred to as the “sick-quitter” effect. As a result, baseline mortality risk in the abstainer group may be artificially inflated, making moderate drinkers appear comparatively healthier and leading to an underestimation of alcohol-related harm [26]. Therefore, although the association with reduced all-cause mortality reaches statistical significance in some analyses, it should be interpreted with caution, as any potential survival benefit does not outweigh the established adverse effects of alcohol on liver disease progression in this population [27].

### Methodological heterogeneity

A key point identified in this review is the considerable methodological heterogeneity across the included studies, both in the definition of "moderate consumption" and in the methods used to measure alcohol intake. The variation in consumption thresholds (ranging from < 20 g/day to 30 g/day for both men and women) reflects a lack of standardization and directly impacts the comparability of results.

Another significant factor is the assessment methodology for alcohol consumption. Most studies relied on self-administered questionnaires or interviews, which are subject to recall bias and underreporting, particularly among individuals with known liver disease. Only one study utilized an objective biomarker, phosphatidylethanol (PEth), which is considered a more sensitive and specific marker for recent alcohol consumption [9]. The lack of standardized assessment methods and the reliance on self-reported measures may explain part of the observed variability and reinforce the need for future studies that combine subjective approaches with biological biomarkers.

Furthermore, the studies analyzed populations with distinct clinical and ethnic profiles, with a predominance of European [7, 9, 11] and Asian [10, 14] samples. This limits the generalizability of the findings to Latin American populations, such as the Brazilian population. This gap highlights the importance of future national investigations into the impact of alcohol on patients with Steatotic Liver Disease, considering the specific genetic and cultural nuances of alcohol consumption in Brazil.

Consistent with these sources of heterogeneity, sensitivity analyses further demonstrated that the results were not entirely stable. The exclusion of individual studies led to meaningful changes in both the magnitude and statistical significance of the effect estimates, indicating that the overall findings were influenced by specific studies. This variability likely reflects differences in study design, population characteristics, and, importantly, alcohol consumption patterns, particularly binge drinking, which was not consistently accounted for across studies.

### Risk of hepatocellular carcinoma (HCC)

The study by Kimura et al. [14] demonstrated that light alcohol consumption, defined as less than 20 g/day, was associated with a significantly increased risk of hepatocellular carcinoma (HCC) in patients with MASLD. These findings reinforce the concept that, in individuals with advanced liver disease, no level of alcohol intake can be considered safe. From a pathophysiological standpoint, even small amounts of alcohol may exert pro-oncogenic effects through multiple mechanisms [28]. Ethanol and its primary metabolite, acetaldehyde, can induce DNA damage and genomic instability, while also activating pro-fibrotic and pro-tumorigenic signaling pathways, including those mediated by transforming growth factor-β (TGF-β), nuclear factor κB (NF-κB), and reactive oxygen species (ROS) [17]. Collectively, these processes promote hepatocyte proliferation and pathological angiogenesis, thereby creating a microenvironment conducive to hepatocarcinogenesis [29].

In addition, the interaction between alcohol exposure and metabolic dysfunction appears to further amplify oncogenic risk. Insulin resistance and the chronic inflammatory state characteristic of metabolic syndrome enhance the expression of inflammatory mediators and hepatic growth factors. When combined with alcohol-induced oxidative stress, these mechanisms may accelerate the progression from steatohepatitis to malignant transformation. Consequently, even light alcohol consumption may have clinically meaningful adverse effects in patients with advanced steatotic liver disease, supporting current recommendations for complete alcohol abstinence in this population, as emphasized by AASLD and EASL guidelines [3, 4].

### Cardiovascular effects and the confounding role of light alcohol consumption

The effect of alcohol on cardiovascular outcomes in patients with steatotic liver disease (SLD) remains controversial. The study by Janjua et al. (2022) [13] showed that light to moderate alcohol consumption was associated with a lower risk of hospitalization for cardiovascular disease, with a reduction of up to 29%, particularly among men consuming 8 to 21 drinks per week. However, this benefit did not extend to cardiovascular mortality and disappeared in individuals with excessive drinking patterns, such as binge drinking (heavy episodic consumption), which proved harmful to cardiovascular health.

The presumed cardioprotective effect of light alcohol consumption has been attributed to improvements in lipid profile (increased HDL and reduced oxidized LDL), decreased platelet aggregation, and enhanced insulin sensitivity [30]. Nevertheless, these potential benefits must be interpreted cautiously, as they are often confounded by lifestyle factors, such as healthier diets and higher levels of physical activity, which independently reduce cardiovascular risk. Thus, part of the observed protective effect may reflect a healthy behavior bias rather than a direct beneficial action of alcohol.

Moreover, the apparent cardiovascular benefit must be weighed against cumulative hepatic risk. The same amount of alcohol that may slightly reduce the risk of cardiac events can worsen liver fibrosis and increase the risk of hepatocellular carcinoma, especially in individuals with insulin resistance and obesity. Therefore, although some cardioprotective effect has been observed in general populations, the risk–benefit balance is unfavorable in patients with MASLD. Current evidence does not support recommending light alcohol consumption as a cardiovascular preventive strategy in individuals with SLD, and abstinence remains the more prudent guidance (3,4).

### Alcohol consumption patterns beyond total intake

An important limitation of this study is the lack of standardized characterization of alcohol consumption patterns across the included studies, which reflects a limitation of the existing literature rather than of this review. Although most studies classified exposure primarily according to average alcohol intake, several also collected more detailed information on drinking behavior, including frequency of consumption and binge drinking, using validated instruments such as AUDIT.

Some studies, included in this review, have been suggested that drinking pattern may influence clinical outcomes independently of total alcohol volume. Blomdahl et al. demonstrated that binge drinking was independently associated with fibrosis progression, with an adjusted odds ratio (aOR) of approximately 5, indicating a markedly increased risk even among individuals with otherwise moderate average alcohol consumption [9]. Similarly, Ekstedt et al. found that episodic heavy drinking was significantly more frequent among patients with fibrosis progression, supporting the role of intermittent high-dose alcohol exposure as a driver of liver disease worsening [11].

Younossi et al. also evaluated binge drinking as a separate exposure and reported increased all-cause mortality independent of total alcohol intake, suggesting that risk cannot be fully captured by average consumption alone. In addition, Janjua et al. observed that the potential cardioprotective effects of moderate alcohol consumption were attenuated or lost in the presence of binge drinking behavior, reinforcing the detrimental impact of this pattern [15].

Other studies addressed binge drinking indirectly through their study design. Chang et al. excluded individuals with binge drinking patterns in order to isolate the effects of regular moderate alcohol consumption, while Ajmera et al. similarly excluded participants with heavy episodic drinking, acknowledging its potential to confound liver-related outcomes [10]. Together, these findings support the notion that binge drinking represents a distinct and clinically relevant exposure that may occur even in individuals whose total weekly alcohol intake falls within conventionally defined moderate limits.

This pattern may lead to greater hepatic injury through mechanisms such as oxidative stress and inflammatory activation, which are not adequately captured by average alcohol consumption metrics alone [31, 32]. However, due to heterogeneity in the definition and reporting of drinking patterns, as well as their frequent use as secondary or exclusion criteria rather than primary exposure variables, it was not possible to perform a pooled analysis stratified by drinking behavior [3]. This variability may have contributed to between-study heterogeneity and may partially explain inconsistencies across findings. Future studies should incorporate standardized and detailed assessments of alcohol consumption patterns to better elucidate their role in MASLD progression.

### Study limitations

Despite the methodology employed, this systematic review has several limitations that should be acknowledged for a critical interpretation of the findings. First, there was substantial heterogeneity among the included studies, particularly in analyses related to liver fibrosis progression (I^2^ > 80% in some meta-analyses). This variability largely stems from the lack of a standardized definition of moderate alcohol consumption, which ranged from 7 to 30 g/day across different cohorts.

Additionally, studies differed in how alcohol intake was measured. Most relied on self-reported questionnaires or interviews, which are subject to underreporting and recall bias, especially among individuals diagnosed with liver disease. Only one study used phosphatidylethanol (PEth), a biomarker considered more reliable for assessing recent alcohol consumption [9]. Among studies using questionnaires, several did not specify which instrument was applied [7, 10, 12, 13, 15]. Ideally, the Alcohol Use Disorders Identification Test (AUDIT), a standardized and validated tool, should have been used consistently in this type of research.

Another limitation is that the assessment of publication bias should be interpreted with caution. All meta-analyses included fewer than 10 studies, which limits the reliability of both funnel plot interpretation and Egger’s test, as recommended by Cochrane guidelines. Therefore, the absence of detected bias does not definitively exclude its presence [33].

Genetic variability may influence susceptibility to alcohol-related liver injury and may partly modify the relationship between alcohol intake and liver outcomes [34]. However, genetic information was not uniformly reported across the included studies. Notably, only one study evaluated PNPLA3 genotyping and performed genotype-stratified sensitivity analyses, which did not show a significant difference in histologic change by baseline drinking status among high-risk carriers [8].

Because the remaining studies did not provide genetic data in a harmonized manner, it was not possible to adjust for or pool the effect of genetic polymorphisms. Therefore, residual confounding by genetic background cannot be excluded, and this limitation may be particularly relevant in populations with different ethnic compositions and different prevalences of risk alleles such as PNPLA3 [35].

Substantial regional variability in alcohol consumption patterns and the burden of liver disease should be considered when interpreting these findings. Global data show marked geographic disparities, with regions such as East Asia bearing a disproportionate share of liver-related morbidity, influenced by factors including viral hepatitis, environmental exposures, and lifestyle changes [36]. In this context, the impact of alcohol is unlikely to be uniform across populations, as differences in drinking behaviors, particularly binge drinking, and interactions with region-specific risk factors may significantly modify outcomes. Therefore, caution is warranted when generalizing these results, and future studies should incorporate regional epidemiological context. [37].

Furthermore, as the review included only observational studies, causal relationships between alcohol consumption and steatotic liver disease progression cannot be established [38]. Finally, there was no uniform stratification regarding fibrosis stage, drinking pattern (regular consumption vs. abstinence), or rigorous control of confounding factors such as diet and physical activity.

These limitations do not invalidate the findings but highlight the need for cautious interpretation and for future studies designed to reduce methodological heterogeneity and improve risk estimation accuracy.

## Conclusions

The results of this systematic review demonstrate that alcohol consumption, even at levels considered light or moderate, is associated with poorer hepatic outcomes in patients with MASLD. We identified a consistent trend toward an increased risk of fibrosis progression and a higher incidence of hepatocellular carcinoma among moderate consumers, particularly those with pre-existing advanced fibrosis or metabolic dysfunction.

Although some studies suggest a potential reduction in all-cause mortality and cardiovascular events among individuals with low alcohol intake, these apparent effects are likely influenced by lifestyle biases and do not offset the negative impact of ethanol on hepatic function. Consequently, the overall balance of evidence suggests that there is no safe level of alcohol consumption for patients with steatotic liver disease, reinforcing the recommendation for total abstinence as the most prudent and effective clinical approach.

The methodological heterogeneity among the included studies and the scarcity of research conducted in Latin American populations represent significant limitations. These gaps highlight the urgent need for future studies with greater standardization and the mandatory use of objective biomarkers (such as PEth) for assessing alcohol intake.

In summary, this review strengthens the evidence base supporting the most recent international guidelines from AASLD and EASL. It underscores that even moderate alcohol consumption is an independent and clinically relevant risk factor for the progression of steatotic liver disease. These findings should serve as a critical resource for improving clinical guidance, enhancing patient counseling, and informing public health policies aimed at preventing liver diseases associated with metabolic dysfunction.

## Supplementary Information

Supplementart Material 1.

## Data Availability

The original contributions presented in this study are included in the article and its supplementary material. Further inquiries regarding the datasets or methodology can be directed to the corresponding author.

## Abbreviations

AASLD: American Association for the Study of Liver Diseases
aOR: Adjusted Odds Ratio
AUDIT: Alcohol Use Disorders Identification Test
AUDIT-C: Alcohol Use Disorders Identification Test-Consumption
BMI: Body Mass Index
CI: Confidence Interval
CVD: Cardiovascular Disease
CYP2E1: Cytochrome P450 2E1
EASL: European Association for the Study of the Liver
HCC: Hepatocellular Carcinoma
HDL: High-Density Lipoprotein
HR: Hazard Ratio
IL-6: Interleukin-6
LDL: Low-Density Lipoprotein
MASLD: Metabolic Dysfunction-Associated Steatotic Liver Disease
MetALD: Metabolic dysfunction-associated steatotic liver disease with increased alcohol intake
NAFLD: Nonalcoholic Fatty Liver Disease
NF-κB: Nuclear Factor kappa B
NOS: Newcastle-Ottawa Scale
OR: Odds Ratio
PEth: Phosphatidylethanol
PNPLA3: Patatin-like phospholipase domain-containing protein 3
PRISMA: Preferred Reporting Items for Systematic Reviews and Meta-Analyses
PROSPERO: International Prospective Register of Systematic Reviews
ROS: Reactive Oxygen Species
SLD: Steatotic Liver Disease
TGF-β: Transforming Growth Factor-beta
TNF-α: Tumor Necrosis Factor-alpha
