# Supplementary material for: Moderate alcohol consumption and clinical outcomes in MASLD: a systematic review and meta-analysis of longitudinal cohorts
Source: BMC Gastroenterol. 2026 Apr 30;26:368. doi: 10.1186/s12876-026-04814-5 (PMC13277244; doi:10.1186/s12876-026-04814-5)
Supplement: Supplementary file 1 — Supplementart Material 1. [file 12876_2026_4814_MOESM1_ESM.docx]

**Supplementary Online Content**

| 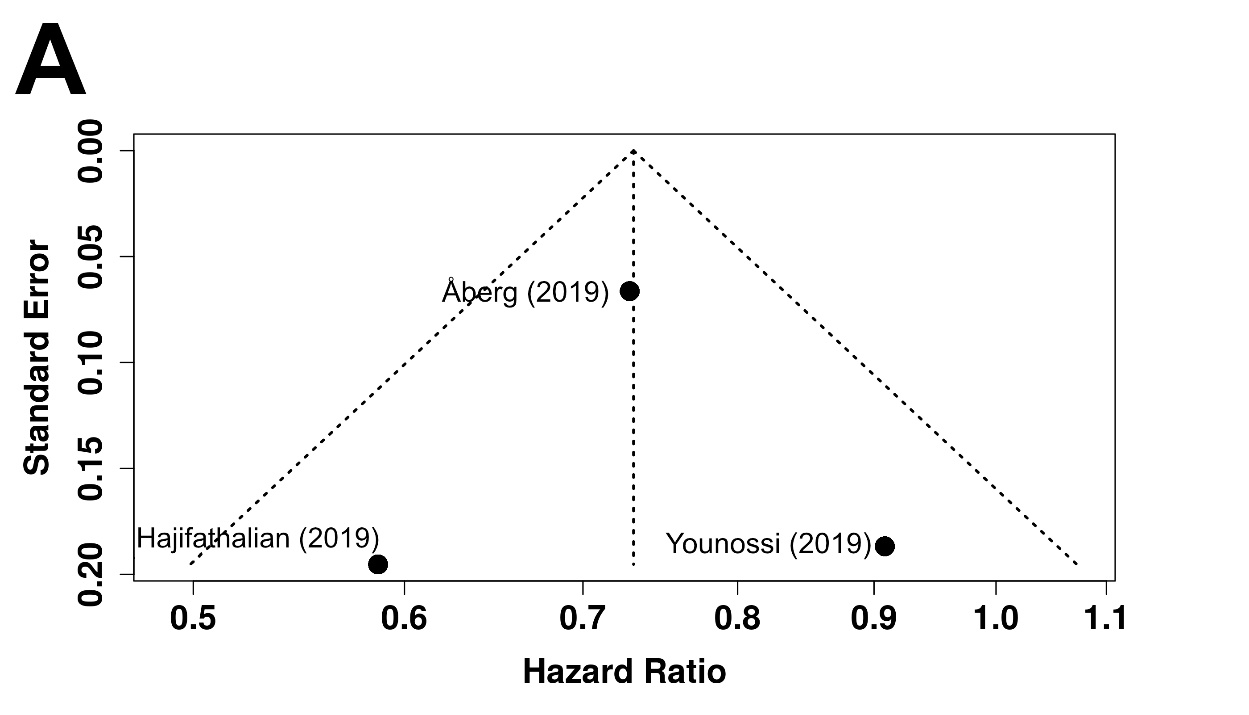 |
| --- |
| 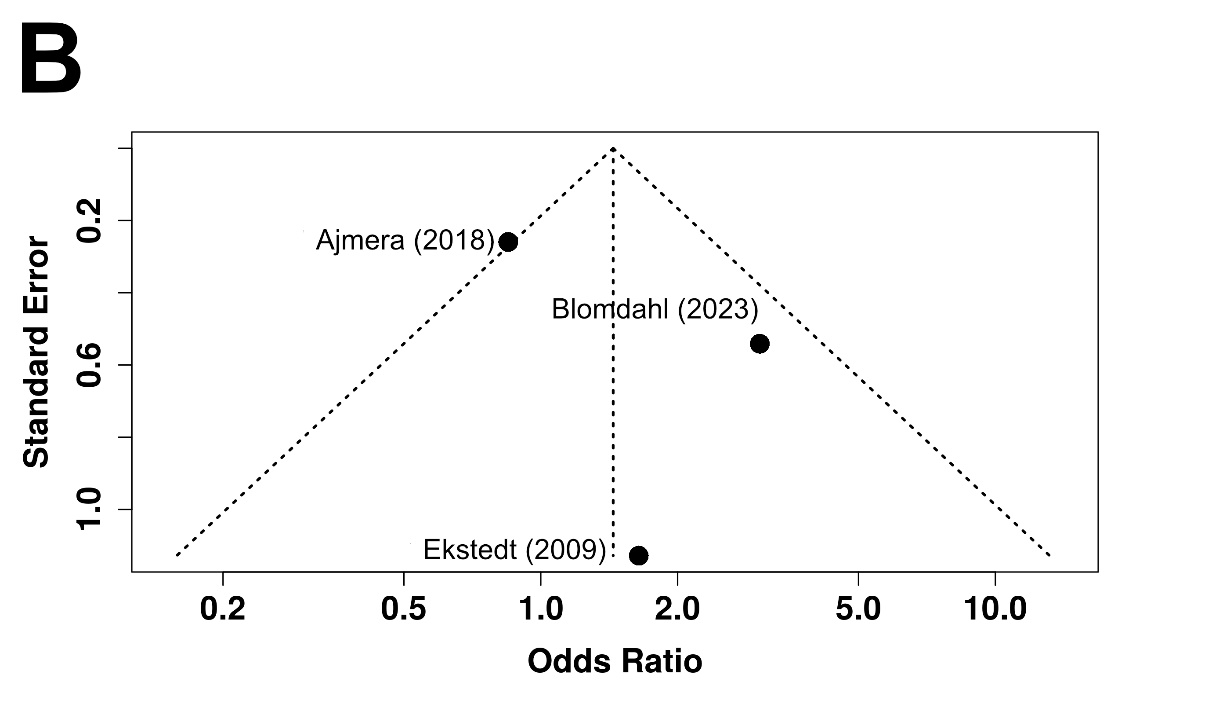 |

# Supplementary Figure S1: Funnel plots for publication bias. No evidence of asymmetry was observed on visual inspection for all-cause mortality (HR) and fibrosis progression (OR), which was confirmed by Egger’s test (p = 0.995 and p = 0.518, respectively). Publication bias was not assessed for fibrosis progression (HR) due to the limited number of studies.
